# Effects of Combined Treatment with Sodium Dichloroacetate and Sodium Valproate on the Genes in Inflammation- and Immune-Related Pathways in T Lymphocytes from Patients with SARS-CoV-2 Infection with Pneumonia: Sex-Related Differences

**DOI:** 10.3390/pharmaceutics16030409

**Published:** 2024-03-16

**Authors:** Donatas Stakišaitis, Linas Kapočius, Vacis Tatarūnas, Dovydas Gečys, Auksė Mickienė, Tomas Tamošuitis, Rasa Ugenskienė, Arūnas Vaitkevičius, Ingrida Balnytė, Vaiva Lesauskaitė

**Affiliations:** 1 Department of Histology and Embryology, Medical Academy, Lithuanian University of Health Sciences, 44307 Kaunas, Lithuania; linas.kapocius@lsmu.lt (L.K.); ingrida.balnyte@lsmu.lt (I.B.); 2 Laboratory of Molecular Oncology, National Cancer Institute, 08660 Vilnius, Lithuania; 3 Institute of Cardiology, Laboratory of Molecular Cardiology, Lithuanian University of Health Sciences, 50161 Kaunas, Lithuania; vacis.tatarunas@lsmu.lt (V.T.); dovydas.gecys@lsmu.lt (D.G.); vaiva.lesauskaite@lsmu.lt (V.L.); 4 Department of Infectious Diseases, Lithuanian University of Health Sciences, 47116 Kaunas, Lithuania; aukse.mickiene@lsmu.lt; 5 Department of Intensive Care Medicine, Lithuanian University of Health Sciences, 50161 Kaunas, Lithuania; tomas.tamosuitis@lsmu.lt; 6 Department of Genetics and Molecular Medicine, Lithuanian University of Health Sciences, 50161 Kaunas, Lithuania; rasa.ugenskiene@lsmu.lt; 7 Institute of Clinical Medicine, Faculty of Medicine, Vilnius University Hospital Santaros Klinikos, Vilnius University, 08661 Vilnius, Lithuania; arunas.vaitkevicius@santa.lt

**Keywords:** SARS-CoV-2 infection, sodium dichloroacetate and sodium valproate combination, investigational medicine, T lymphocytes, sex-related differences, inflammation

## Abstract

The study presents data on the anti-inflammatory effects of a combination of sodium dichloroacetate and sodium valproate (DCA–VPA) on the expression of inflammation- and immune response-related genes in T lymphocytes of SARS-CoV-2 patients. The study aimed to assess the effects of DCA–VPA on the genes of cytokine activity, chemokine-mediated signaling, neutrophil chemotaxis, lymphocyte chemotaxis, T-cell chemotaxis, and regulation of T-cell proliferation pathways. The study included 21 patients with SARS-CoV-2 infection and pneumonia: 9 male patients with a mean age of 68.44 ± 15.32 years and 12 female patients with a mean age of 65.42 ± 15.74 years. They were hospitalized between December 2022 and March 2023. At the time of testing, over 90% of sequences analyzed in Lithuania were found to be of the omicron variant of SARS-CoV-2. The T lymphocytes from patients were treated with 5 mmol DCA and 2 mmol VPA for 24 h in vitro. The effect of the DCA–VPA treatment on gene expression in T lymphocytes was analyzed via gene sequencing. The study shows that DCA–VPA has significant anti-inflammatory effects and apparent sex-related differences. The effect is more potent in T cells from male patients with SARS-CoV-2 infection and pneumonia than in females.

## 1. Introduction

The discovery of novel therapeutic indications for authorized medicines and the development of new medicinal products by a combination of known drugs for treating severe infections are areas of significant interest in medicine. The research presents data from the anti-inflammatory effects of sodium dichloroacetate (DCA) and sodium valproate (VPA) salt combination (DCA–VPA) on SARS-CoV-2 infection patients’ T lymphocytes in vitro. VPA and DCA are well-established medicines with a therapeutic experience and known safety profile, dosage, and blood levels. Therefore, they are attractive for studies to explore potential new therapeutic indications for these medicines or their combination. DCA–VPA has anti-inflammatory effects in mouse thymocytes, significantly affecting the expression of inflammation-related genes involved in thymocyte inflammation-related cytokine activity, the inflammatory response, and the IL17 signaling pathways [1].

Severe acute respiratory syndrome coronavirus 2 disease (SARS-CoV-2 infection; COVID-19) disrupts the immune response, leading to high levels of cytokine and other inflammation molecules released in the blood. However, cytokine-inhibitor therapies have been unsuccessful and can even aggravate the patient’s condition in some cases [2,3]. Severe infections are a sign of an inflammatory and immune response to infection and a manifestation of severe mitochondrial metabolic disturbances [4,5,6], which can lead to an unfavorable outcome and death [7,8,9]. Therefore, treating metabolic disorders is a vital target for treating serious SARS-CoV-2 [10].

The meta-analysis shows that the risk of SARS-CoV-2 infection is the same in men and women. Men with COVID-19 are three times more frequently admitted to the intensive care unit and also more often dying [11,12,13,14]. In contrast to women, a higher mortality in COVID-19 men is found in all age groups [13,15]. Male gender is a decisive predisposing factor, identified by multiple analyses of the patient, for severe SARS-CoV-2 infection [11].

DCA and VPA are investigational drugs exhibiting anti-inflammatory effects in viral and bacterial infections, and these effects are associated with the reversal of mitochondrial dysregulation and impaired immune responses [16,17]. Regulatory guidelines for investigational medicines research call for the importance of the impact of sex on the assessment of drug efficacy, and it is essential to provide relevant information on the sex-specific efficacy of drugs under development [18,19]. Female and male cells have different responses to chemicals. Studies on the sex-specific effects of drugs must be addressed in preclinical and clinical trials [20,21].

DCA has been studied as an investigational medicine for over 50 years. It is a preparation found to lower blood glucose levels; it works as an inhibitor of pyruvate dehydrogenase kinase (PDK) activity, increasing the activity of pyruvate dehydrogenase (PDH) and its complex (PDHC); and it is commonly used to treat diseases associated with mitochondrial defects and increased congenital or acquired lactic acid production [22]. The inactivation of the PDHA1 subunit is associated with metabolic reprogramming and the overproduction of lactic acid, a pro-inflammatory metabolite that increases the production of pro-inflammatory cytokines [23]. PDH inactivation during severe infectious inflammation and sepsis is detected in peripheral blood mononuclear cells, macrophages, and vascular endothelial cells [24,25]. As a modulator of mitochondria, DCA is also a potential agent for inhibiting inflammation. Elevated blood lactate concentration is a marker of the severity of SARS-CoV-2 infection and an independent predictor of poor prognosis in COVID-19, sepsis, and other infections [26,27]. 

VPA may reduce the expression of the ACE2 receptor and the transmembrane serine protease 2 (TMPRSS2) receptor, which requires co-expression for SARS-CoV-2 viral entry into a cell [28,29]. Male cells have a single ACE2 allele, as the ACE2 gene is encoded on the X chromosome [30,31]. The female cell’s mosaicism of the X chromosome is associated with the heterogeneity of the ACE2 allele, which may encode partial resistance to COVID-19 infection in women [32]. Female tissues have lower ACE2 expression than males [33,34,35]. Older women have lower ACE2 activity than younger women, which is uncommon in men [35]. Testosterone increases ACE2 levels, while estrogens suppress ACE2 expression [36,37,38]. No sex-related differences in TMPRSS2 expression were found in human and mouse lungs [39]. Pharmacological intervention with an androgen receptor antagonist significantly inhibited ACE2 expression in the lungs of male mice [39]. TMPRSS2 is potentially the most promising target for SARS-CoV-2 infection therapy as its specific expression is in alveolar cells [40,41]; TMPRSS2 inhibitors are effective against SARS-CoV-2 [28]. VPA downregulates TMPRSS2 expression [41,42]. SARS-CoV-2 infection was reduced by more than 75% in patients treated with VPA monotherapy compared to the general population [43]. Based on clinical and laboratory endpoints, VPA-treated patients develop less severe COVID-19 than control patients [44].

Bioinformatics approaches allow a broader analysis of the potential effects of registered drugs, thus opening up the possibility of their use in treating SARS-CoV-2 infection [45]. One such drug is VPA, a histone deacetylase (HDAC) inhibitor [46]. The opening of the SARS-CoV-2 spike trimer receptor binding domains is blocked by VPA and coenzyme A conjugate [47]. The X-ray crystal structure of SARS-CoV-2 has shown that central protease (M^pro^, 3CL^pro^) is a crucial protein in the viral life cycle [48,49,50]. HDAC inhibitors bind tightly to the active site of the crystallographic structure of viral M^pro^ [51]. Viral M^pro^ is a target for antiviral drugs [52,53]. The SARS-CoV-2 protease NSP5 interacts with HDAC2. It is thought that NSP5 may inhibit HDAC2 translocation into the nucleus and affect HDAC2 potency and mechanisms of inflammation [54,55]. HDAC2 binding to promoters was lower in females than males [56]. VPA reduced HDAC2 levels in the frontal cortex tissue of male rats, but there was no such effect in females [57]. HDAC2 protein levels are sensitive to the selective effects of VPA [58]. VPA has been investigated in treating various viral infections [45,59]. VPA is being investigated as an immunomodulator [45,60].

Elevated glucose levels favor the progression of SARS-CoV-2 infection [61]; glycolysis is essential for the rapid growth and proliferation of virus-activated T cells [62,63,64]. Treatment with DCA or VPA monotherapy reduces blood glucose levels [22,65,66]. 

The novelty of the DCA–VPA research is based on the mechanisms of synergistic effects of these agents. VPA activates the SLC5A8 gene through DNA demethylation by inhibiting HDAC activity [67]. The SLC5A8 carries short-chain fatty acids and DCA into cells [68,69]. SLC5A8, participating in the mitochondrial β-oxidation pathway, enhances the transport of DCA into the cell, thereby altering the regulation of mitochondrial metabolism [70,71]. SLC5A8 is well expressed in cells in the kidney [72] and lung [73] tissues, which are targets of SARS-CoV-2. Thus, the synergistic effect of VPA and DCA may be pharmacologically relevant to investigate the efficacy of SARS-CoV-2 infection treatment.

Investigating sex-specific differences in treating SARS-CoV-2 infection may lead to a new approach to the personalized treatment of the disease. This study aimed to evaluate DCA–VPA as a potential investigational drug for the treatment of SARS-CoV-2 infection, to elucidate the possible biological pathways of the pathogenesis of the disease, to examine the impact of sex-specific pharmacological mechanisms of SARS-CoV-2 infection, and to explore the possibility of an individualized treatment of the disease. 

The study aimed to investigate the effect of the DCA–VPA treatment on the expression of inflammation- and immune response-related genes in T lymphocytes of SARS-CoV-2-infected patients with pneumonia. The objectives of the study were to determine the effect of the DCA–VPA treatment on cytokine activity, chemokine-mediated signaling, neutrophil chemotaxis, lymphocyte chemotaxis, T-cell chemotaxis, and regulation of T-cell proliferation pathways, and to detect sex-related differences in the treatment efficacy.

The study showed that the treatment of T lymphocytes from SARS-CoV-2-infected patients with pneumonia with DCA–VPA in vitro had an anti-inflammatory effect, manifested by inhibiting pro-inflammatory gene expression in T lymphocytes. The impact on cytokine activity, chemokine-mediated signaling, neutrophil chemotaxis, lymphocyte chemotaxis, and regulation of T-cell proliferation pathways was superior in male T lymphocytes than in female T lymphocytes, and gender-related differences in treatment effect were identified.

## 2. Materials and Methods

### 2.1. The Investigational Medicinal Product

The investigational medicinal product combines sodium dichloroacetate (DCA; Sigma-Aldrich, Steinheim, Germany) and sodium valproate (VPA; Sigma-Aldrich, Steinheim, Germany). The combination of these medicinal products is a pending patent filed by us that covers DCA–VPA as a new medicinal product for the treatment of viral and bacterial infections (National patent application No. LT2023 532; 22 August 2023), as well as for the treatment of cancer (Official bulletin of the state patent bureau of the Republic of Lithuania, No. 6874, filling date 17 April 2020, https://vpb.lrv.lt/uploads/vpb/documents/files/VPB-OB-Nr23-2021-12-10-1d.pdf (accessed on 2 February 2024)); a European patent application has been submitted (European patent application No. 21168796.7, filing date 16 April 2021, https://register.epo.org/application?number=EP21168796 (accessed on 2 February 2024)). 

### 2.2. Patients with SARS-CoV-2 Infection and Pneumonia

The study included 21 patients (9 males and 12 females) hospitalized in an infectious diseases unit due to a serious condition caused by COVID-19 infection and pneumonia. The study was approved by the Kaunas Regional Biomedical Research Ethics Committee (Authorization to conduct biomedical research 2021-12-28; No. BE-2-140). Patients were hospitalized at the Department of Infectious Diseases of the Lithuanian University of Health Sciences Kaunas Hospital (Kaunas, Lithuania). Blood was collected from the patients, and T lymphocytes were isolated. Patients were enrolled according to established inclusion and exclusion criteria. Participants could be included in the study only if all criteria were applied. Adult males and females who independently gave informed consent to participate in the study were selected. 

Inclusion criteria were as follows: (1) adult male and female patients, older than 18 years; (2) patients who understand the essence of the study and can independently give written informed consent; (3) RT-PCR detected a positive SARS-CoV-2 sample (naso–pharyngeal swab); (4) patients with new pneumonia diagnosed via X-ray and according to the criteria of COVID-19 guidance; (5) no active malignancy; (6) no history of pancreatitis; and (7) no prior valproic acid use. 

The exclusion criteria were as follows: (1) patients with acute myocardial infarction or stroke; (2) patients with severe hepatic or renal impairment; (3) known HIV positivity, active, uncontrolled infection (e.g., hepatitis A, B, or C infection); (4) patients taking immunosuppressants; (5) cancer patients; (6) patients undergoing blood purification therapy (hemodialysis, hemofiltration, hemadsorption, plasma exchange, plasma adsorption, or peritoneal dialysis). 

Criteria for severe illness were as indicated in the guidelines (https://www.covid19treatmentguidelines.nih.gov/overview/clinical-spectrum (accessed on 2 February 2024) when pneumonia was associated with at least one of the following symptoms: individuals who have a SpO_2_ < 94% on room air at sea level, a ratio of arterial partial pressure of oxygen to fraction of inspired oxygen (PaO_2_/FiO_2_) < 300 mm Hg, a respiratory rate > 30 breaths/min, or lung infiltrates > 50%.

T lymphocytes were obtained from 9 men aged 68.44 ± 15.32 years and 12 women aged 65.42 ± 15.74 years hospitalized from December 2022 to March 2023. At that time, the omicron variant of SARS-CoV-2 was detected in >90% of sequences tested in Lithuania (https://ourworldindata.org/covid-cases (accessed on 2 February 2024). In the female group, the time between a positive SARS-CoV-2 PCR test and blood sampling was 3.27 ± 2.00 days, and in the male group, it was 4.38 ± 3.28 days. The blood glucose concentration on the day of blood sampling was 6.38 ± 1.36 mM/L in males and 9.01 ± 3.12 mM/L in females; the female blood glucose concentration was slightly elevated due to the presence of 5 patients with type 2 diabetes mellitus in the study group.

### 2.3. Isolation of T Lymphocytes from Human Venous Blood by Ficoll-Paque™ Density Gradient and Treatment

Patient blood was taken using standard venipuncture in 10 mL vacuum tubes with anticoagulant KEDTA. Mononuclear cells were purified using the Ficoll-Paque™ gradient centrifugation method according to the manufacturer’s instructions (Cytiva, Uppsala, Sweden). Commercial PBS GIBCO, a phosphate-buffered saline (LifeTechnologies, The Hague, The Netherlands) and HBSS, a balanced salt solution (Sigma-Aldrich, Dorset, UK), were purchased. All reagents were prewarmed to room temperature before use (18–20 °C). The blood was diluted 1 to 1 in PBS. In total, 20 mL (4 mL per tube) of Ficoll-Paque™ was added to another 5 centrifuge tubes (15 mL). The diluted blood was then slowly layered onto the Ficoll-Paque™ layer using a Pasteur pipette. The tubes were then centrifuged at 532× *g* for 15 min at room temperature. The cell-containing interface layer was then drawn into new 15 mL centrifuge tubes. The cell washing procedure was repeated twice. The cells were suspended carefully using a sterile Pasteur pipette in approximately 3 volumes (depending on the estimated volume of cells in a tube) of HBSS and centrifuged at 354× *g* for 5 min at room temperature. The cells were resuspended in HBSS (approximately 3 volumes) and centrifuged at room temperature at 244 g for 5 min. The cells were then resuspended in HBSS. Cell viability was assessed with trypan blue dye using the Evos XL Core Imaging System (ThermoFisherScientific, Carlsbad, CA, USA). The T lymphocytes isolated from patients were treated with DCA–VPA (5 mM DCA and 2 mM VPA) for 24 h in vitro. Due to the synergistic effect of DCA and VPA, the dose of sodium dichloroacetate chosen for the combination was half that of our monotherapy to treat the other cells [74].

### 2.4. Total RNA Extraction and Next-Generation Sequencing

A commercial PureLink™ RNA Purification Kit (Invitrogen™, Thermo Fisher Scientific, Bleiswijk, The Netherlands) was used to extract total RNA from lymphocytes. After extraction, total RNA was quantified using a NanoDrop 2000 spectrophotometer (Thermo Scientific, Waltham, MA, USA), and the RNA integrity number (RIN) was evaluated on the Agilent 2100 capillary electrophoresis system (Agilent Technologies, Santa Clara, CA, USA) using an RNA 6000 Nano kit (Agilent Technologies, Santa Clara, CA, USA). Samples with RIN > 8.5 were used in downstream next-generation sequencing (NGS) experiments. According to the manufacturer’s manual, NGS library preparation was performed using the QIAseq targeted RNA Inflammation and Immunity Transcriptome panel kit (Qiagen, Hilden, Germany). As a starting RNA input, 400 ng of RNA was used for each sample. The final PCR amplification stage was performed in a 22-cycle setting. The size ranges of NGS libraries were measured with a High Sensitivity DNA 1000 kit (Agilent Technologies, Santa Clara, CA, USA) on an Agilent 2100 device. For final library quantification, the Qubit™ High Sensitivity dsDNA Quantification Assay kit (Invitrogen™, Thermo Fisher Scientific, Bleiswijk, The Netherlands) was applied, and fluorescence measurements were taken on a Qubit 4.0 fluorometer (Invitrogen™, Thermo Fisher Scientific, Bleiswijk, The Netherlands). Libraries were denatured and diluted according to the NextSeq library denaturation and dilution guide. A sequencing run was performed on the Illumina NextSeq 550 sequencer (Illumina, San Diego, CA, USA) using the 150-cycle Illumina High Output Kit v2.5 (Illumina, San Diego, CA, USA) under default conditions. T lymphocyte samples from males and females were sequenced on the same flow cell.

### 2.5. Bioinformatic Analysis

The quality of the data was assessed using MultiQC v1.13 [75]. Adapter sequences with 3 nucleotides and sequences shorter than 15 nucleotides, as well as sequences with a quality score lower than 25, were removed using Cutadapt v1.9.1 [76]. The human genome (GRCh38.p13) was downloaded from the Ensembl database [77]. The remaining sequences were aligned to the human genome using the STAR 2.1.3 tool [78]. The gene expression matrix was obtained using FeatureCounts v3.15 [79]. The sequence expression was normalized using the upper quartile method, and genes with a total expression across samples less than 50 were removed. A differential gene expression analysis was performed using DESeq2 v3.15 [80], and *p*-values were adjusted using the Benjamin–Hochberg method. An enrichment analysis of biological pathways was conducted using the DAVID server [81,82]. This normalization allows for a fair comparison between gene sets of different sizes. Data from treated T lymphocytes were compared with untreated cells. The gene expression data from treated and control conditions were normalized and logarithmically transformed. The enrichment analysis results from the gene set enrichment analysis (GSEA) were analyzed using the Enrichplot v1.2 and ClusterProfiler v4.8.2 R packages [83]. GSEA was performed using predefined algorithms to calculate enrichment scores and *p*-values for each dominant pathway or gene set. Gene set annotations were obtained from gene ontology (GO), KEGG, and Reactome databases. NES is the primary statistic for identifying gene sets significantly overrepresented in a ranked list of genes. It is derived from the ES (Enrichment Score) but is normalized for the size of each gene set. The impact of the DCA–VPA treatment was calculated by comparing the gene expression in treated and control T cells. Significant changes in gene expression were determined when *p* < 0.05.

## 3. Results

### 3.1. Data on the Effect of the DCA–VPA Treatment on the Expression of Genes Related to Inflammation and Immune Response in T Lymphocytes from Men with SARS-CoV-2 Infection and Pneumonia

The treatment with DCA–VPA of T lymphocytes of male patients suffering from SARS-CoV-2 infection and pneumonia significantly reduced the expression of 40 genes related to inflammation and immune response and involved in cytokine activity, chemokine mediated signaling, neutrophil chemotaxis, lymphocyte chemotaxis, and the regulation of T-cell proliferation pathogenic mechanisms after 24 h of treatment. Table 1 shows the male patients’ T lymphocyte gene expression data (Log_2_ counts average of control and treated cells), Log_2_ fold change compared to control and treated cells data, and *p*-value for each gene in the tested group. 

**Figure 1:**
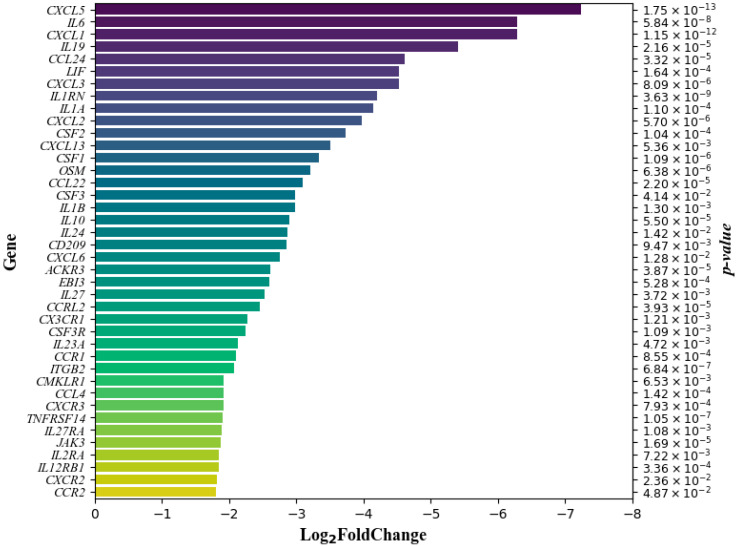
Differentially expressed genes in men suffering from SARS-CoV-2 infection with pneumonia: T lymphocytes following DCA–VPA treatment.

The DCA–VPA treatment significantly downregulated the expression of *ACKR3*, *CCL22*, *CCL24*, *CCL4*, *CCR1*, *CCR2*, *CCRL2*, *CD209*, *CMKLR1*, *CSF1*, *CSF2*, *CSF3*, *CSF3R*, *CX3CR1*, *CXCL1*, *CXCL13*, *CXCL2*, *CXCL3*, *CXCL5*, *CXCL6*, *CXCR2*, *CXCR3*, *EBI3*, *IL10*, *IL12RB1*, *IL19*, *IL1A*, *IL1B*, *IL1RN*, *IL24*, *IL23A*, *IL27*, *IL27RA*, *IL2RA*, *IL6*, *ITGB2*, *JAK3*, *LIF*, *OSM*, and *TNFRSF14* genes of T lymphocytes of males suffering from SARS-CoV-2 infection with pneumonia. The strength of the effect of DCA–VPA suppression on gene expression is shown in Figure 1.

The bar chart represents the fold change in gene expression on a logarithmic scale (log_2_FoldChange). The direction of the data, as indicated by the negative values on the *x*-axis, suggests that the genes listed are downregulated following treatment with DCA–VPA in T lymphocytes. In this context, a negative log_2_ fold change means that the expression of these genes is lower in the treated cells compared to a control group. The greater the absolute value of the log_2_ fold change, the more significant the downregulation. The *p*-values provided alongside each gene indicate the statistical significance of the downregulation, with lower *p*-values denoting higher statistical significance.

GO analysis revealed that differentially expressed changes in control and DCA–VPA-treated T lymphocyte genes of men with SARS-CoV-2 infection are involved in pathogenic mechanisms of inflammation pathways of interest. Table 2 shows the DCA–VPA treatment’s effects on T lymphocyte genes involved in cytokine activity, chemokine-mediated signaling, neutrophil chemotaxis, lymphocyte chemotaxis, and regulation of T-cell proliferation pathways in men suffering from SARS-CoV-2 infection with pneumonia. 

The treatment of male T lymphocytes with DCA–VPA significantly reduced the gene expression of 22 pro-inflammatory cytokines involved in the cytokine activity pathway. These genes are *CCL22*, *CCL24*, *CSF1*, *CSF2*, *CSF3*, *CXCL1*, *CXCL2*, *CXCL3*, *CXCL5*, *CXCL6*, *CXCL13*, *EBI3*, *IL1A*, *IL1B*, *IL1RN*, *IL6*, *IL10*, *IL19*, *IL24*, *IL27*, *LIF*, and *OSM*. Twenty-two genes were affected in the cytokine activity pathway, with an enrichment score of −0.82 and an NES of −2.73, indicating a substantial underrepresentation of these genes following DCA–VPA treatment. The *p*-value of 1.00 × 10^−10^ signifies a very high statistical significance.

DCA–VPA-treated male T lymphocytes showed a significantly decreased expression of 17 pro-inflammatory genes, namely, *ACKR3*, *CCL4*, *CCL22*, *CCL24*, *CCR1*, *CCR2*, *CCRL2*, *CMKLR1*, *CX3CR1*, *CXCL1*, *CXCL2*, *CXCL3*, *CXCL5*, *CXCL6*, *CXCL13*, *CXCR2*, and *CXCR3*, which are involved in the chemokine-mediated signaling pathway. This pathway had 17 genes downregulated, with an enrichment score of −0.68, an NES of −2.08, and a *p*-value of 6.110^−5^, reinforcing these changes’ statistical significance. The affected genes are mainly chemokine receptors and ligands, suggesting a reduced signaling activity in chemotaxis.

The DCA–VPA treatment significantly reduced the expression of the pro-inflammatory chemokine in the neutrophil chemotaxis pathway genes *CCL4*, *CCL22*, *CCL24*, *CSF3R*, *CXCL1*, *CXCL2*, *CXCL3*, *CXCL5*, *CXCL6*, *CXCL13*, and *ITGB2* in men’s T lymphocytes. There were 11 genes with decreased expression in this category, with an enrichment score of −0.63, an NES of −1.86, and a *p*-value of 0.0015, implying a diminished recruitment or movement of neutrophils.

When analyzing the expression of genes in the lymphocyte chemotaxis pathway, it was found that the DCA–VPA treatment reduced the expression of five pro-inflammatory chemokine genes in male T lymphocytes. The genes affected by the treatment were *CCL4*, *CCL22*, *CCL24*, *CXCL13*, and *CXCR3*. A smaller set of five genes showed downregulation in this pathway, with an enrichment score and an NES of −0.68 and −1.53, respectively. The *p*-value of 0.036 suggests a statistically significant change, indicating that the treatment may hinder lymphocyte migration.

**Figure 2:**
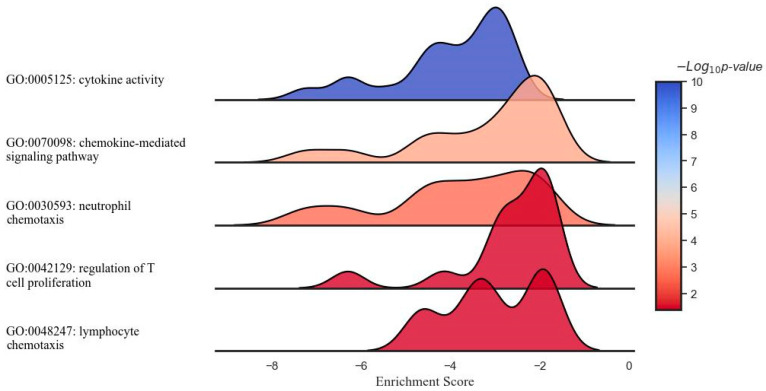
Ridge plot representing the gene set enrichment analysis of inflammation- and immune-related response modulation through DCA–VPA treatment in male patients with SARS-CoV-2 infection and pneumonia.

The DCA–VPA treatment significantly reduced the gene expression of the regulation of T-cell proliferation pathway factors. These genes were *CCR2*, *CD209*, *EBI3*, *IL1A*, *IL1B*, *IL2RA*, *IL6*, *IL10*, *IL12RB1*, *IL23A*, *IL27*, *IL27RA*, *JAK3*, and *TNFRSF14*. The expression of 15 genes related to T-cell proliferation was considerably decreased, as indicated by an enrichment score of −0.47, an NES of −1.52, and a *p*-value of 0.048. Figure 2 shows the results of the gene set enrichment analysis (GSEA) illustrating the suppressing effect of the DCA–VPA treatment on specific sets of inflammation- and immune response-related genes in cytokine activity, chemokine mediated signaling, neutrophil chemotaxis, lymphocyte chemotaxis, and regulation of T-cell proliferation pathways in T lymphocytes from men with SARS-CoV-2 infection. 

Figure 2 shows the results of a GSEA illustrating the modulatory effect of the DCA–VPA treatment on specific sets of inflammation- and immune response-related genes in T lymphocytes from men with SARS-CoV-2 infection. Each plot represents a unique gene ontology (GO) term associated with distinct aspects of response. From top to bottom, the gene sets represent suppressing effects of cytokine activity, chemokine-mediated signaling, neutrophil chemotaxis, regulation of T cell proliferation, and lymphocyte chemotaxis biological pathways. The x-axis denotes the ES, which reflects the degree to which a set of genes is overrepresented at the extremes (top or bottom) of the entire ranked list of genes. The y-axis represents the gene sets, organized by inflammation- and immune-related function categories. The color intensity within each plot corresponds to the −Log10 *p*-value, indicating the level of statistical significance, with blue indicating a higher significance level. The peaks shifting towards the left of the 0 mark suggest the downregulation of the corresponding gene sets post treatment with DCA–VPA, which may imply a modulating effect on these inflammation/immune mechanisms, potentially leading to a suppression of responses. Table 3 shows the changes in the expression of genes involved in cytokine activity, chemokine-mediated signaling, neutrophil chemotaxis, lymphocyte chemotaxis, and lymphocyte chemotaxis in T lymphocytes from women with SARS-CoV-2 infection, which were significantly altered in the 24 h after treatment with DCA–VPA when comparing the untreated to the treated group.

### 3.2. Data on the Effect of the DCA–VPA Treatment on the Expression of Genes Related to Inflammation and Immune Response in T Lymphocytes from Females with SARS-CoV-2 Infection with Pneumonia

**Figure 3:**
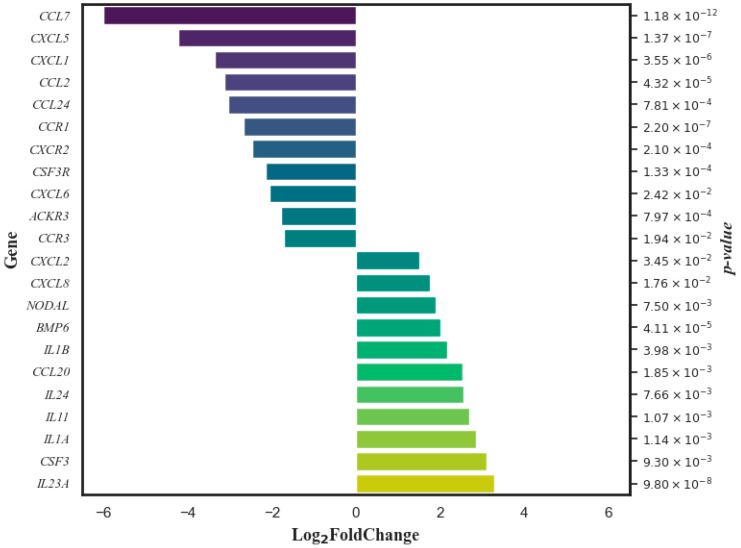
Genes whose expression in women’s T lymphocytes was significantly changed by DCA–VPA treatment in patients suffering from SARS-CoV-2 infection with pneumonia.

The DCA–VPA treatment of SARS-CoV-2-infected women’s T lymphocytes significantly altered the expression of 21 genes, including 11 genes—*ACKR3*, *CCL2*, *CCL7*, *CCL24*, *CCR1*, *CCR3*, *CSF3R*, *CXCL1*, *CXCL5*, *CXCL6*, and *CXCR2*—whose expression was decreased, and 10 genes—*BMP6*, *CCL20*, *CXCL2*, *CXCL8*, *IL11*, *IL1A*, *IL1B*, *IL23A*, *IL24*, and *NODAL*—whose expression was increased. The DCA–VPA treatment’s efficacy in gene expression changes is illustrated in Figure 3.

The Log_2_ fold change data are organized in such a way that genes with reduced expression appear in descending order of change magnitude, and those with increased expression are in ascending order. The *p*-value on the right assesses the statistical significance of these changes.

Table 4 extends these findings to the GO analysis of DCA–VPA-treated female T lymphocytes, identifying genes in the biological pathways of cytokine activity, chemokine-mediated signaling, neutrophil chemotaxis, and lymphocyte chemotaxis. The table details the number of genes involved, the enrichment score, the NES, the effects on gene expression, and the significance of these effects as denoted by the *p*-values.

The DCA–VPA treatment altered the expression of 11 genes in the cytokine activity pathway. *CSF3* was downregulated, while *BMP6*, *CCL20*, *CXCL2*, *CXCL8*, *IL11*, *IL1A*, *IL1B*, *IL23A*, *IL24*, and *NODAL* were significantly upregulated. An enrichment score of 0.41, an NES of 1.74, and a *p*-value of 0.015 indicate a significant shift toward enhanced cytokine activity.

Within the chemokine-mediated signaling pathway, the expression of 10 genes was significantly decreased: *ACKR3*, *CCL2*, *CCL24*, *CCL7*, *CCR1*, *CCR3*, *CXCL1*, *CXCL5*, *CXCL6*, and *CXCR2*. This is supported by an enrichment score of −0.61, an NES of −1.89, and a *p*-value of 0.025, indicating a notable suppression of chemokine-mediated signaling.

For the neutrophil chemotaxis pathway, the expression of *CCL2*, *CCL24*, *CCL7*, *CSF3R*, *CXCL1*, *CXCL5*, *CXCL6*, and *CXCR2* was significantly downregulated, as shown by an enrichment score of −0.66, an NES of −1.97, and a *p*-value of 0.001, denoting a significant reduction in neutrophil chemotaxis by the treatment.

The treatment’s effects in the lymphocyte chemotaxis pathway were evident in the downregulation of three genes, *CCL2*, *CCL7*, and *CCL24*, with an enrichment score of −0.72, an NES of −1.55, and a *p*-value of 0.04, indicating diminished lymphocyte chemotaxis.

**Figure 4:**
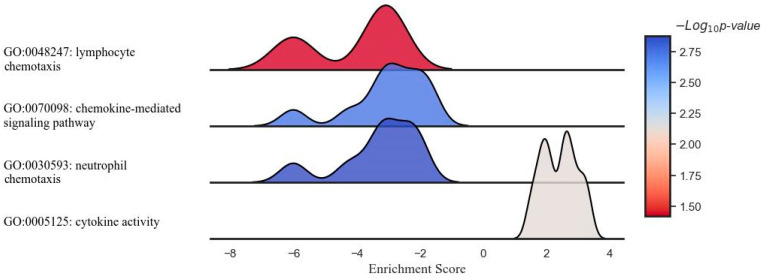
Ridge plot representing the gene set enrichment analysis of inflammation- and immune-related response modulation in pathways of interest by DCA–VPA treatment in female patients with SARS-CoV-2 infection and pneumonia.

The GO analysis indicated no significant effect of the DCA–VPA treatment on the regulation of T-cell proliferation pathway in women with SARS-CoV-2 infection, suggesting that the treatment under the conditions studied did not impact this particular aspect of T-cell function. Figure 4 shows the GSEA results, illustrating the effect of the DCA–VPA treatment on specific sets of inflammation- and immune response-related genes in cytokine activity, chemokine-mediated signaling, neutrophil chemotaxis, and lymphocyte chemotaxis pathways in T lymphocytes from women with SARS-CoV-2 infection and pneumonia. 

In a study elucidating the molecular impact of the DCA–VPA treatment in female patients with SARS-CoV-2 infection, a ridge plot was employed to visualize the gene set enrichment analysis across several key immunological pathways. The image presents a ridge plot illustrating the GSEA for four different gene ontology (GO) pathways in the context of female patients with SARS-CoV-2 infection treated with DCA–VPA. The plot visualizes the distribution of enrichment scores across the four pathways. It correlates these scores with the statistical significance of the enrichment, as indicated by the negative logarithm of the *p*-value (−Log_10_ *p*-value). The enrichment scores on the horizontal axis revealed no strong up- or downregulation in the cytokine activity pathway, with the associated *p*-values suggesting minimal statistical significance. A slight negative shift in the neutrophil chemotaxis pathway hinted at a potential gene downregulation, though the significance remained low. The leftward distribution in the chemokine-mediated signaling pathway was more pronounced, indicating a more substantial downregulation of genes, with a corresponding moderate increase in statistical significance. Most notably, the lymphocyte chemotaxis pathway demonstrated a marked negative enrichment, signaling a robust downregulation of gene activity, corroborated by the highest statistical significance among the evaluated pathways. This comprehensive GSEA underscores the nuanced and differential gene expression modulation enacted by the DCA–VPA treatment, offering insights into the therapeutic alteration of inflammation- and immune response-related pathways of SARS-CoV-2 infection.

### 3.3. Sex-Specific Gene Expression Responses to DCA-VPA Treatment in T Lymphocytes of SARS-CoV-2-Infected Patients with Pneumonia

**Figure 5:**
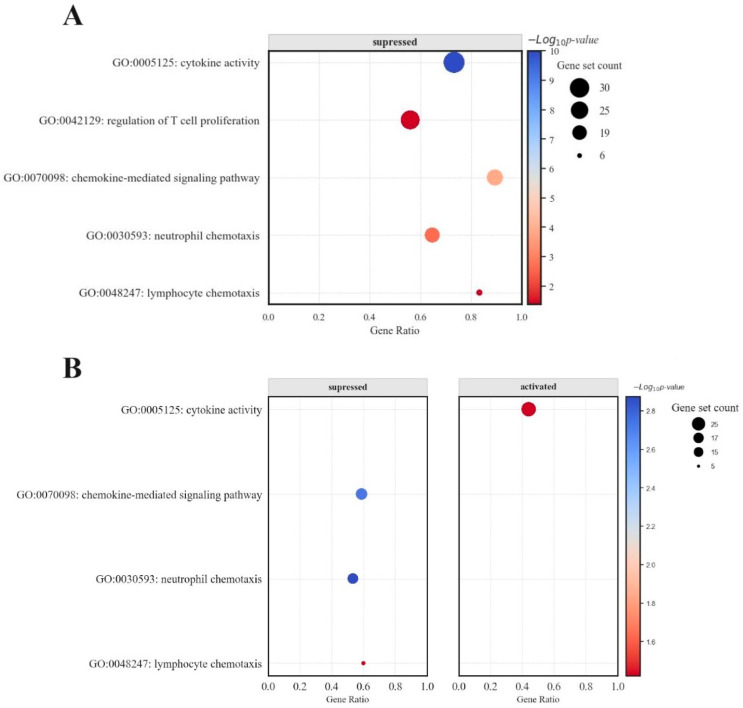
Bubble plot representing the impact of DCA–VPA therapy on inflammation- and immune-related gene sets (**A**) in males with SARS-CoV-2 infection and pneumonia patients, and (**B**) in females with SARS-CoV-2 infection and pneumonia patients.

The analysis of DCA–VPA-treated patients’ T lymphocytes revealed distinct gene expression profiles, highlighting a differential sex-specific response to the treatment in biological pathways of concern. The DCA–VPA treatment significantly altered the expression of 22 genes in male T lymphocytes and 11 genes in the female cells’ cytokine activity pathway. In the chemokine-mediated signaling pathway, the expressions of 17 genes expressions were altered in males and 10 in females; 11 genes were changed in neutrophil chemotaxis in males and 8 in females; 5 genes were altered in lymphocyte chemotaxis in males; and 3 genes were changed in T lymphocytes in females. These figures also indicate sex-related differences. The GO analysis showed that the DCA–VPA treatment did not significantly affect the regulation of T-cell proliferation pathway in SARS-CoV-2-infected women. In contrast, substantial changes in 14 genes were detected in male cells. Figure 5 represents the bubble plot impact of the DCA–VPA therapy on inflammation- and immune-related gene sets in the investigated biological pathways.

In male patients, gene sets associated with cytokine activity and chemokine-mediated signaling pathways were downregulated, as evidenced by negative enrichment scores in GSEA. Conversely, female patients exhibited a bimodal distribution in the enrichment scores for lymphocyte chemotaxis, indicating a nuanced regulatory effect of the treatment on this pathway. While still affected, the chemokine-mediated signaling pathway showed a less pronounced response than male cells. Additionally, the bubble (dot) plots for females demonstrated a balance between upregulated and downregulated genes, a pattern not as evident in male patients. The bar chart data for women showed a more evenly distributed expression change across the examined genes, including significant upregulation in specific genes—a response not observed in the male cohort.

## 4. Discussion

The study on the effects of DCA–VPA showed sex-related differences in cytokine activity, chemokine signaling, the regulation of neutrophil chemotaxis and T lymphocyte proliferation, and in the expression of inflammation- and immunity-associated genes in the T lymphocytes of men and women with SARS-CoV-2 infection with pneumonia.

### 4.1. DCA–VPA Effects on Cytokine Activity and Sex-Related Differences 

Notably, the bar charts revealed a considerable downregulation in genes related to cytokine activity in males, with 22 genes showing substantial log2 fold changes. This pattern was not as prominent in females, where the changes in gene expression were more varied and included notable upregulation instances. Treatment with DCA–VPA significantly reduced the expression of *IL1A*, *IL1B*, *IL1RN*, *IL6*, *IL10*, *IL19*, *IL24*, *IL27*, *OSM*, and *LIF* in the T lymphocytes of male patients with infection. The DCA–VPA treatment of T lymphocytes from male SARS-CoV-2-infected patients with pneumonia was probably most effective in suppressing the expression of the IL6 gene, a key cytokine of the infection. IL6 levels are significantly elevated in the blood of patients with severe COVID-19 [84]. IL6 has been identified as a cause of the cytokine release syndrome in SARS-CoV-2 infection [85]. The *S* protein of SARS-CoV-2 increased *IL6* mRNA expression in macrophages [86]. In COVID-19 patients, a decrease in *IL6* mRNA expression indicates a reduction in inflammatory reactions and gradual recovery [87]. IL6 inhibitors are being used to treat severe COVID-19 [85]. DCA–VPA inhibited OSM (oncostatin M) and LIF gene expression in male T lymphocytes of SARS-CoV-2 patients. The positive regulator of IL6 signaling, OSM, was upregulated in lethal COVID-19 patients and SARS-CoV-2-infected patients with moderate compared to mild pneumonia [88]. Monocytes and neutrophils produced OSM, stimulating IL6 production in fibroblasts and endothelial cells. The level of circulating OSM is positively correlated with the severity of COVID-19 [89]. LIF cytokine, a member of the IL6 family, is highly expressed in lung cells of mild and severe COVID-19 patients but not in healthy subjects. LIF is elevated in the lungs of ARDS patients [90]. *EBI3* (Epstein–Barr virus-induced gene 3) is a subunit of the composite cytokines IL27 and IL35; EBI3 activity was linked to its capacity to mediate IL6 *trans*-signaling, and by mediating *trans*-signaling, it can promote pro-inflammatory IL6 functions [91].

DCA–VPA suppressed *IL1* in male SARS-CoV-2 patients’ T lymphocytes. IL1 is essential in developing severe SARS-CoV-2 infection associated with mortality [92]. *IL1A* is expressed in lung cells [93], and it predicts a bad prognosis for COVID-19 [94]. IL1A and IL1B are associated with a susceptibility to pandemic A/H1N1 influenza virus [95]. Although the overall effect of DCA–VPA on the cytokine release pathway is inhibitory (see Figure 3), the fact that the combination also inhibits the expression of the *IL1RN* may make the effect of our treatment ambiguous. IL1RN modulates the COVID-19 cytokine release syndrome via endogenous “anti-inflammatory” pathways. IL1RN variants modulate the severity of SARS-CoV-2 infection: the IL1RN CTA haplotype and the rs419598 CC single nucleotide variant are associated with lower levels of pro-inflammatory IL1B, IL6, and IL2, together with higher levels of anti-inflammatory IL-10. They are associated with a significant reduction in male mortality. We did not identify genetic variants in IL1RN, which does not allow us to determine the genetic regulation of the inflammatory pathway by IL1RN variants. However, other studies suggest that IL1 is an essential target for further studies in severe SARS-CoV-2 infection [92].

The suppressive effect of DCA–VPA on the gene expression of the IL10 family of cytokines in male T lymphocytes would also indicate an inhibitory effect on SARS-CoV-2 infection. Patients with severe COVID-19 have elevated plasma levels of the IL10 family of cytokines, IL19, and IL24, which decrease during recovery [96]. Airway epithelial cells secrete IL19 in response to stimulation with various pro-inflammatory cytokines [97]. The plasma and saliva levels of IL19 significantly increase in patients with severe COVID-19 [98]. To generalize regarding IL10 gene expression change and the characterization of the treatment’s effects on male T lymphocytes, it is crucial to emphasize that the gene set enrichment analysis (GSEA) showed significant downregulation in cytokine gene expression, including both pro- and anti-inflammatory cytokines, with a notable negative net enrichment score (NES) of −2.73 (*p*-value 1.00 × 10^−^^10^). This broad spectrum modulation suggests the treatment’s complex impact on cytokine dynamics rather than a unilateral effect. Specifically, including IL10, a traditionally anti-inflammatory cytokine, in the list of downregulated genes highlights the intricate balance the drug achieves in cytokine regulation. The therapeutic potential of the treatment, as suggested by our analysis, may stem from its capacity to attenuate excessive inflammatory responses, a key pathological feature of severe COVID-19, rather than simply augmenting or suppressing individual cytokines. The nuanced understanding of cytokine interactions, including the pleiotropic effects of cytokines like IL10, supports our conclusion of the treatment’s net anti-inflammatory effect, contributing to its efficacy in managing COVID-19-related inflammation.

The data that DCA–VPA suppresses *IL27* expression in male T lymphocytes may also be important for treating infection. IL27 at the time of admission was strongly associated with patients with severe/critical COVID-19 pneumonia and is, therefore, an excellent predictor of adverse prognosis [94], although there is conflicting evidence that IL27 levels were significantly higher in survivors of the severe form of COVID-19 than in those who died from the severe form [99].

DCA–VPA significantly downregulated *CSF1*, *CSF2*, and *CSF3* expression in the cytokine activity pathway. High granulocyte colony-stimulating factor (G-CSF) levels are detected in parallel with elevated serum levels of IL6 and IL10 in severe COVID-19 [100,101]. T cells can activate monocytes via the pro-inflammatory CSF2 and CSF1 receptors and induce a cytokine storm [102]. *CSF3* is the most highly upregulated gene in SARS-CoV-2 infection, indicating that CSF3 may be a candidate target for drug therapy [103].

The treatment of male T lymphocytes with DCA–VPA significantly reduced the expression of chemokines genes as *CCL4*, *CCL22*, *CCL24*, *CCR1*, *CCR2*, *CCRL2*, *CX3CR1*, *CXCL1*, *CXCL13*, *CXCL2*, *CXCL3*, *CXCL5*, *CXCL6*, *CXCR2*, and *CXCR3.* Chemokines are critical mediators of inflammation, which remove pathogens. However, the overproduction of chemokines promotes inflammation: chemokines are a direct contributor to acute respiratory syndrome, which is fatal in around 40% of severe cases. Chemokines are involved in various stages of SARS-CoV-2 infection [104]. CCL22 is a biomarker of severe COVID-19; CCL22 controls immunity by promoting the communication of regulatory T cells to dendritic cells, which is essential for recruiting Th2 cells in the respiratory tissues [105]. COVID-19 patients who progressed showed persistent Th2 inflammation, which was strongly affected by CCL24 [106]. The genes for the vital pro-inflammatory factors CXCL1, CXCL2, CXCL3, CXCL5, and CSF2 are targets of SARS-CoV-2 infection [107]. Studies in mice have shown that the deactivation of ACE2 leads to an increase in the release of pro-inflammatory CXCL1 and CXCL5 and an increase in neutrophil infiltration with inflammatory lung injury [84]. MERS-CoV or SARS-CoV-2 viruses modulate the expression of *CXCL5* and *CXCL6* in lung cells. These genes encode proteins that affect lymphocyte and neutrophil function [108,109]. Deleting the *CXCL5* in a mouse model reduced lung inflammation, indicating that CXCL5 inhibition may be a target to control lung inflammation [109]. As COVID-19 patients progressed, a statistically significant dysregulation of CXCL5 was found [110]. The CXCL6 exerts its chemotactic effect by interacting with receptors CXCR1 and CXCR2 to attract neutrophil granulocytes [108]. Pro-inflammatory CXCL13 has a high specificity for disease progression: it was significantly higher in ICU than in non-ICU patients. CXCL13 is implicated in pulmonary fibrosis and the regulation of B-cell response [111].

In the T lymphocytes of SARS-CoV-2-infected women with pneumonia, the DCA–VPA treatment changed the expression of 11 genes: it increased the expression of *IL11*, *IL1A*, *IL1B*, *IL23A*, *IL24*, *CCL20*, *CXCL2*, *CXCL8*, *BMP6*, and *NODAL*, and decreased the expression of *CSF3*. IL11, a member of the IL6 cytokine family, is defined as an “epithelial interleukin”. High levels of IL11 are linked to prolonged inflammation [112]. IL23A is one of two subunits of cytokine IL23. IL23 is produced by dendritic cells and macrophages and is an integral part of the inflammatory response [113].

The CCL20 chemokine binds to the CCR6 receptor, which determines the chemotaxis of dendritic cells, T lymphocytes, and B cells [114]. Increased CCL20 expression is correlated with increased circulating CCL20 levels during the prolonged course of COVID-19 and with prolonged viral clearance [115]. CXCL8 is a prognostic marker for the severity of SARS and MERS infection. During SARS-CoV-2 infection, CXCL8 levels are increased in blood and alveoli; the increase is also associated with bacterial infection [104]. The increased CXCL8 expression is associated with anti-inflammatory effects. The CXCL8 enhances T helper cell function [116].

BMP6 (bone morphogenetic protein 6) is involved in antiviral response, enhances the antiviral activity of IFNs, and regulates the expression of critical antiviral effectors [117,118]. The NODAL gene encodes the TGF-β (transforming growth factor-β) protein. TGF-β is essential in the control of immunity and inflammation mechanisms [119].

The comparison of the enrichment analysis of cytokine activity in the T lymphocytes of male and female SARS-CoV-2-infected patients treated with DCA–VPA shows sex differences in the modulatory effect. The enrichment score is shifted in opposite directions, with males to the left and females to the right of the midline (0 value); the Log10 *p*-value indicates a higher level of statistical significance for female T lymphocytes. In male T lymphocytes, 22 genes associated with inflammation are suppressed, whereas in female T lymphocytes, only CSF3 is suppressed (as in male T lymphocytes). *BMP6*, *CXCL20*, *CXCL8*, *IL1A*, *IL1B*, *IL11*, *IL23A*, *IL24*, and *NODAL* were found to be activated in female cells. The increased *BMP6*, *CXCL8* and *CXCL20* expressions are associated with anti-inflammatory effects. Whether the increase in IL11 levels is pathogenic or a natural host response to restore homeostasis in disease remains unanswered [112]. *NODAL* is important in controlling immunity and inflammation mechanisms, and its expression could be linked to female-specific immune responses. The same could be interpreted as the determined opposite effect of DCA–VPA in the increase of *IL1A*, *IL1B*, *IL23A*, *IL24*, and *CXCL2* expression after the treatment of female cells. The difference in female immune mechanisms from those of males in SARS-CoV-2 infection is reported [120]. Our studies have shown that the effect of DCA–VPA on male T lymphocytes is significantly associated with the suppression of *IL6* and related cytokines genes, which is not the case in female cells. Here, we examine the sex-specific anti-inflammatory effects of DCA–VPA by assessing T lymphocyte gene expression changes in the chemokine-mediated signaling, neutrophil chemotaxis, lymphocyte chemotaxis, and regulation of T-cell proliferation pathways.

### 4.2. DCA–VPA Effects on Chemokine-Mediated Signaling, Neutrophil Chemotaxis, Lymphocyte Chemotaxis, and Regulation of T-Cell Proliferation Pathways and Sex-Related Differences 

The suppression of *CCL22*, *CCL24*, *CXCL1*, *CXCL2*, *CXCL3*, *CXCL5*, *CXCL6*, and *CXCL13* in male T lymphocytes coincides with the overlap between cytokine-activated and chemokine-mediated signaling pathways, suggesting that the DCA–VPA treatment is associated with an anti-inflammatory effect via both of these pathways. No overlap of genes inhibited by the DCA–VPA treatment in the cytokine activity and chemokine-mediated signaling pathways was determined in female T lymphocytes. 

In men and women patients with SARS-2-CoV-2 infection, the treatment of T lymphocytes with DCA–VPA significantly decreased the expression of the same genes—*ACKR3*, *CCL24*, *CCR1*, *CXCL1*, *CXCL5*, and *CXCL6*—in the chemokine-mediated signaling pathway. ACKR3 (Atypical Chemokine Receptor 3) in lymphocytes separates two phenotypically, transcriptionally, and functionally distinct populations of mouse MZ B cells [121]. Its relevance to inflammation and SARS-CoV-2 infection has not been investigated. SARS-CoV-2 upregulates CCR1 and CCR2 in human thoracic dorsal root ganglia, indicating an inflammatory mediator effect on pulmonary sensory neurons. Thus, the pharmacological inhibition of receptors would suggest that it could potentially suppress hyperinflammation in critically ill COVID-19 patients [104].

In male T lymphocytes, sex-specific effects of the DCA–VPA treatment, which significantly suppressed *CCL4*, *CCL22*, *CCR2*, *CCRL2*, *CMKLR1*, *CX3CR1*, *CXCL2*, *CXCL3*, *CXCL13*, and *CXCR3* expression, were revealed in the chemokine-mediated signaling pathway. Severe COVID-19 patients over-expressed *CCL4*, and lung macrophages showed an over-expression of the gene [122]. *CCRL2* mRNA and protein were detected in monocytes, macrophages, neutrophils, CD4- and CD8-positive T lymphocytes, B cells, monocyte-derived dendritic cells, and CD34-positive cells [123]. CCRL2 was increased by proinflammatory stimuli, such as LPS or TNF-α alone or in combination with IFN-γ or GM-CSF [123,124].

The clinical relevance of CMKLR1 as a marker of lung inflammation in ARDS was confirmed using RNA sequencing data, which showed that *CMKLR1* expression is significantly increased in lung monocytes and macrophages in COVID-19 patients. CMKLR1-targeted PET is essential for monitoring the dynamics of lung inflammation and response to anti-inflammatory therapy [125]. The pro-inflammatory chemokine receptors CCR1, CCR2, CXCR3, and CX3CR1 mediate the immune response in the lung and are present in myeloid cells, T cells, and NK cells [126]. CXCR3 is the receptor for CXCL10. The CXCL10-CXCR3 signaling axis is in the pathogenesis of severe COVID-19 infection, making it a potential therapeutic target [127].

In female T lymphocytes, sex-specific effects of the DCA–VPA treatment, which involved the significant suppression of *CCL24*, *CCL7*, *CCR3*, and *CXCL1* were determined in the chemokine-mediated signaling pathway. Severe COVID-19 patients were reported to over-express *CCL7* in lung macrophages [128,129]. The CCR3 receptor is involved in migrating macrophages, NK cells, and monocytes and is upregulated in early post-SARS-CoV-2 infection [104].

In the neutrophil chemotaxis pathway, the DCA–VPA treatment significantly suppressed the expression of *CCL24*, *CCL7*, *CSF3R*, *CXCL1*, *CXCL5*, and *CXCL6* in both male and female T lymphocytes. The following sex-related difference was found: only in men was the expression of *CCL4*, *CCL22*, *CXCL2*, *CXCL3*, *CXCL13*, and *ITGB2* additionally suppressed, while in women, the expression of *CCL2*, *CCL7*, and *CXCR2* was significantly suppressed sex-specifically. 

In the regulation of the T-cell proliferation pathway, the significant downregulation of pro-inflammatory *CCR2*, *CD209*, *EBI3*, *IL1A*, *IL1B*, *IL2RA*, *IL6*, *IL10*, *IL12RB1*, *IL23A*, *IL27*, *IL27RA*, *JAK3*, and *TNFRSF14* was determined in T lymphocytes of men suffering from SARS-CoV-2 infection. However, no reliable gene therapy effect was found in female T lymphocytes in regulating the T-cell proliferation pathway. In addition to the genes mentioned above in the article, in male T cells, the DCA–VPA treatment significantly inhibited *IL12RB1* (interleukin 12 receptor beta 1 subunit), which is linked to a significant association between susceptibility to SARS-CoV-2 infection [130], *IL27RB1*, whose expression is required for CD4^+^ and CD8^+^ T-cell differentiation in humans [131], *JAK3*, which has a cell type-specific role in IL-2-induced proliferative signal transduction [132], and *TNFRSF14* (tumor necrosis factor-related cytokine LIGHT (TNFSF14), which has proinflammatory activity, with multifaceted roles in stimulating T cells [133]. In turn, suppressing *CD209* by the medicinal product could inhibit the receptor for SARS-CoV-2 from attachment onto host cells [134]. Given the complexity of T-cell proliferation processes, sex-related differences in the effects of the DCA–VPA treatment on the regulation of the T-cell proliferation pathway can be discussed in the context of the overall impact on T-cell proliferation [135]. T lymphocytes proliferate, and their functional responses depend on metabolism through T-cell differentiation processes and on the inflammatory microenvironment in which they are located [135]. The inhibition of pro-inflammatory cytokine generation may be linked to the effect of not only DCA but also the inhibition of glycolysis, which may influence the T-cell proliferation pathway in T lymphocytes. 

This study faces a situation where the effect of DCA–VPA is not controlled by an anti-inflammatory agent that modulates inflammatory immune responses via mitochondrial metabolic mechanisms. This could be seen as a limitation of the study. However, this happens because no analogous anti-inflammatory preparation corrects metabolic disturbances. Nevertheless, it is considered that the efficacy of DCA–VPA has been sufficiently demonstrated in the treated cells compared to untreated controls. Another limitation of the study could be that post-menopausal women were studied. As sex-related differences were found, it would be essential to study the T lymphocytes of young women. A pharmacovigilance alert has been issued that patients with COVID-19 should avoid treatment with the anti-inflammatory ibuprofen as it may worsen the condition [136]. Ibuprofen is a specific SLC5A8 blocker [137]. Potentially promising research data have been reported for the treatment of COVID-19 with anti-inflammatory small molecule Ebselen derivative compounds, which are inhibitors of SARS-CoV-2 protease M^pro^, papain-like protease (P^Lpro^), and nsp14 guanine N7-methyltransferase [138]. The actuality and the rationale for applying the anti-inflammatory effects of the DCA–VPA combination study is that VPA activates the *SLC5A8* via DNA demethylation [139]. SLC5A8 carries short-chain fatty acids and transports DCA into cells [68,69]. SLC5A8 transports DCA into the cell [22,70,71], correcting mitochondrial metabolism in tissue cells, and the DCA–VPA treatment should attenuate the inflammation and tissue damage and protect against disease progression. DCAs and VPAs have sex-specific metabolism and biological effects [16,140]. Therefore, further research on the sex-specific efficacy of the preparation is relevant.

## 5. Conclusions

The gene sequencing analysis revealed a significant effect of DCA–VPA on changes in the expression of the genes in cytokine activity, chemokine-mediated signaling, neutrophil chemotaxis, lymphocyte chemotaxis, T-cell chemotaxis, and regulation of T-cell proliferation pathways. DCA–VPA exerts anti-inflammatory effects, and there were apparent sex-related differences: the anti-inflammatory effect was more strongly expressed in T lymphocytes from male patients than female patients with SARS-CoV-2 infection with pneumonia. To further clarify sex differences, it would be appropriate to determine whether these differences are influenced by data on women with diabetes.

The sex-specific modulation of inflammation/immune response by the DCA–VPA treatment in the T lymphocytes of SARS-CoV-2 patients with pneumonia underscores the potential for personalized therapeutic approaches. The distinct immune profiles warrant further investigation into the molecular mechanisms driving these differences and could provide insights into the optimal management of SARS-CoV-2 infection in diverse patient populations.

## 6. Patents

The combination of these medicinal products is a pending patent filed by us that covers DCA–VPA as a new medicinal product for the treatment of viral and bacterial infections (National patent application No. LT2023 532; 22 August 2023), as well as for the treatment of cancer (Official bulletin of the state patent bureau of the Republic of Lithuania, No. 6874, filling date 17 April 2020, https://vpb.lrv.lt/uploads/vpb/documents/files/VPB-OB-Nr23-2021-12-10-1d.pdf (accessed on 2 February 2024); a European patent application has been submitted (European patent application No. 21168796.7, filing date 16 April 2021, https://register.epo.org/application?number=EP21168796 (accessed on 2 February 2024). 

## Figures and Tables

**Table 1:** Gene expression data for T lymphocytes in male patients with SARS-CoV-2 infection in the study groups.

Gene	Log_2_ Fold Change	Gene Expression Change	Log_2_ Count Average of Control Cells	Log_2_ Count Average of Treated Cells	*p* Value
*ACKR3*	−2.61	↓	7.78	6.67	3.87 × 10^−^^5^
*CCL22*	−3.10	↓	8.21	6.78	2.20 × 10^−^^5^
*CCL24*	−4.61	↓	11.70	8.63	3.32 × 10^−^^5^
*CCL4*	−1.92	↓	14.76	14.08	1.42 × 10^−^^4^
*CCR1*	−2.10	↓	12.41	10.88	8.55 × 10^−^^4^
*CCR2*	−1.80	↓	12.94	11.78	4.87 × 10^−^^2^
*CCRL2*	−2.45	↓	9.41	8.09	3.93 × 10^−^^5^
*CD209*	−2.85	↓	7.09	4.84	9.47 × 10^−^^3^
*CMKLR1*	−1.92	↓	9.09	8.09	6.53 × 10^−^^3^
*CSF1*	−3.34	↓	9.71	8.22	1.09 × 10^−^^6^
*CSF2*	−3.73	↓	7.00	4.91	1.04 × 10^−^^4^
*CSF3*	−2.98	↓	6.64	6.08	4.14 × 10^−^^2^
*CSF3R*	−2.24	↓	12.90	11.30	1.09 × 10^−^^3^
*CX3CR1*	−2.27	↓	10.19	8.42	1.21 × 10^−^^3^
*CXCL1*	−6.29	↓	13.00	8.51	1.15 × 10^−^^12^
*CXCL13*	−3.51	↓	4.34	3.34	5.36 × 10^−^^3^
*CXCL2*	−3.98	↓	14.08	11.91	5.70 × 10^−^^6^
*CXCL3*	−4.52	↓	10.63	7.74	8.09 × 10^−^^6^
*CXCL5*	−7.23	↓	15.28	9.89	1.75 × 10^−^^13^
*CXCL6*	−2.75	↓	7.43	6.23	1.28 × 10^−^^2^
*CXCR2*	−1.81	↓	10.43	9.06	2.36 × 10^−^^2^
*CXCR3*	−1.91	↓	8.81	7.96	7.93 × 10^−^^4^
*EBI3*	−2.60	↓	5.75	4.89	5.28 × 10^−^^4^
*IL10*	−2.90	↓	7.88	6.81	5.50 × 10^−^^5^
*IL12RB1*	−1.85	↓	8.69	7.66	3.36 × 10^−^^4^
*IL19*	−5.40	↓	6.69	3.84	2.16 × 10^−^^5^
*IL1A*	−4.14	↓	11.66	9.43	1.10 × 10^−^^4^
*IL1B*	−2.98	↓	16.01	14.87	1.30 × 10^−^^3^
*IL1RN*	−4.21	↓	12.46	9.74	3.63 × 10^−^^9^
*IL23A*	−2.14	↓	9.45	9.33	4.72 × 10^−^^3^
*IL24*	−2.87	↓	10.20	9.16	1.42 × 10^−^^2^
*IL27*	−2.53	↓	5.98	4.25	3.72 × 10^−^^3^
*IL27RA*	−1.89	↓	9.49	8.48	1.08 × 10^−^^3^
*IL2RA*	−1.85	↓	9.60	9.21	7.22 × 10^−^^3^
*IL6*	−6.29	↓	11.31	7.22	5.84 × 10^−^^8^
*ITGB2*	−2.08	↓	14.09	12.86	6.84 × 10^−^^7^
*JAK3*	−1.88	↓	10.76	9.77	1.69 × 10^−^^5^
*LIF*	−4.53	↓	7.88	4.76	1.64 × 10^−^^4^
*OSM*	−3.21	↓	8.58	6.38	6.38 × 10^−^^6^
*TNFRSF14*	−1.91	↓	13.19	12.40	1.05 × 10^−^^7^

**Table 2:** Effect of DCA–VPA treatment on men suffering from SARS-CoV-2 infection with pneumonia: T lymphocyte gene expression in the inflammatory pathways of interest.

Pathway ID	Number of Genes	Enrichment Score	NES	*p* Value	Treatment Has a Significant Effect on the Expression of a Gene
GO:0005125: Cytokine activity	22	−0.82	−2.73	1.00 × 10^−^^10^	Decreased: *CCL22*, *CCL24*, *CSF1*, *CSF2*, *CSF3*, *CXCL1*, *CXCL2*, *CXCL3*, *CXCL5*, *CXCL6*, *CXCL13*, *EBI3*, *IL1A*, *IL1B*, *IL1RN*, *IL6*, *IL10*, *IL19*, *IL24*, *IL27*, *LIF*, *OSM*
GO:0070098: Chemokine-mediated signaling pathway	17	−0.68	−2.08	6.10 × 10^−^^5^	Decreased: *ACKR3*, *CCL4*, *CCL22*, *CCL24*, *CCR1*, *CCR2*, *CCRL2*, *CMKLR1*, *CX3CR1*, *CXCL1*, *CXCL2*, *CXCL3*, *CXCL5*, *CXCL6*, *CXCL13*, *CXCR2*, *CXCR3*
GO:0030593: Neutrophil chemotaxis	11	−0.63	−1.86	1.50 × 10^−^^3^	Decreased: *CCL4*, *CCL22*, *CCL24*, *CSF3R*, *CXCL1*, *CXCL2*, *CXCL3*, *CXCL5*, *CXCL6*, *CXCL13*, *ITGB2*
GO:0048247: Lymphocyte chemotaxis	5	−0.68	−1.53	3.60 × 10^−^^2^	Decreased: *CCL4*, *CCL22*, *CCL24*, *CXCL13*, *CXCR3*
GO:0042098: Regulation of T-cell proliferation	14	−0.47	−1.52	4.10 × 10^−^^2^	Decreased: *CCR2*, *CD209*, *EBI3*, *IL1A*, *IL1B*, *IL2RA*, *IL6*, *IL10*, *IL12RB1*, *IL23A*, *IL27*, *IL27RA*, *JAK3*, *TNFRSF14*

**Table 3:** Gene expression data for T lymphocytes in female patients with SARS-CoV-2 infection and pneumonia in the study groups.

Gene	Log_2_ Fold Change	Gene Expression Change	Log_2_ Count Average of Control Cells	Log_2_ Count Average of Treated Cells	*p* Value
*ACKR3*	−1.79	↓	8.29	6.42	7.97 × 10^−^^4^
*BMP6*	2.02	↑	8.31	10.51	4.11 × 10^−^^5^
*CCL2*	−3.12	↓	15.12	10.06	4.32 × 10^−^^5^
*CCL20*	2.53	↑	11.32	13.39	1.85 × 10^−^^3^
*CCL24*	−3.04	↓	14.04	9.32	7.81 × 10^−^^4^
*CCL7*	−6.00	↓	12.10	5.33	1.18 × 10^−^^12^
*CCR1*	−2.67	↓	13.62	10.25	2.20 × 10^−^^7^
*CCR3*	−1.71	↓	7.47	5.23	1.94 × 10^−^^2^
*CSF3*	3.10	↑	6.58	9.35	9.30 × 10^−^^3^
*CSF3R*	−2.15	↓	13.24	10.89	1.33 × 10^−^^4^
*CXCL1*	−3.36	↓	13.29	9.97	3.55 × 10^−^^6^
*CXCL2*	1.51	↑	12.31	13.66	3.45 × 10^−^^2^
*CXCL5*	−4.22	↓	15.61	10.21	1.37 × 10^−^^7^
*CXCL6*	−2.06	↓	9.39	7.24	2.42 × 10^−^^2^
*CXCL8*	1.77	↑	16.81	18.50	1.76 × 10^−^^2^
*CXCR2*	−2.46	↓	11.26	8.86	2.10 × 10^−^^4^
*IL11*	2.70	↑	0.76	3.54	1.07 × 10^−^^3^
*IL1A*	2.85	↑	9.39	11.86	1.14 × 10^−^^3^
*IL1B*	2.17	↑	14.61	16.62	3.98 × 10^−^^3^
*IL23A*	3.30	↑	9.09	11.97	9.80 × 10^−^^8^
*IL24*	2.55	↑	9.12	11.02	7.66 × 10^−^^3^

**Table 4:** Effect of DCA–VPA treatment on gene expression in biological pathways in the T lymphocytes of women suffering from SARS-CoV-2 infection with pneumonia.

Pathway ID	Number of Genes	Enrichment Score	NES	*p* Value	Significant Effect of Treatment on Gene Expression
GO:0005125: Cytokine activity	11	0.41	1.74	0.015	Decreased: *CSF3* Increased: *BMP6*, *CCL20*, *CXCL2*, *CXCL8*, *IL11*, *IL1A*, *IL1B*, *IL23A*, *IL24*, *NODAL*
GO:0070098: Chemokine-mediated signaling pathway	10	−0.61	−1.89	0.025	Decreased: *ACKR3*, *CCL2*, *CCL24*, *CCL7*, *CCR1*, *CCR3*, *CXCL1*, *CXCL5*, *CXCL6*, *CXCR2*
GO:0030593: Neutrophil chemotaxis	8	−0.66	−1.97	0.001	Decreased: *CCL2*, *CCL24*, *CCL7*, *CSF3R*, *CXCL1*, *CXCL5*, *CXCL6*, *CXCR2*
GO:0048247: Lymphocyte chemotaxis	3	−0.72	−1.55	0.04	Decreased: *CCL2*, *CCL7*, *CCL24*
GO:0042098: T-cell proliferation					No effect of treatment on genes

## Data Availability

The data presented in this study are available on request from the corresponding author.
